# A practical guide to the implementation of AI in orthopaedic research—Part 5: Data management

**DOI:** 10.1002/jeo2.70581

**Published:** 2025-12-17

**Authors:** Bálint Zsidai, Felix Oettl, James A. Pruneski, Gergely Pánics, Philipp W. Winkler, Eric Hamrin Senorski, Michael T. Hirschmann, Yinan Yu, Robert Feldt, Kristian Samuelsson, ESSKA Artificial Intelligence Working Group

**Affiliations:** ^1^ Sahlgrenska Sports Medicine Center Gothenburg Sweden; ^2^ Department of Orthopaedics, Institute of Clinical Sciences, Sahlgrenska Academy University of Gothenburg Gothenburg Sweden; ^3^ Department of Orthopaedics Skåne University Hospital Malmö/Lund Sweden; ^4^ Department of Orthopaedic Surgery, Balgrist University Hospital University of Zürich Zurich Switzerland; ^5^ Department of Orthopaedic Surgery Tripler Army Medical Center Honolulu Hawaii USA; ^6^ Budapesti Uzsoki Street Hospital Budapest Hungary; ^7^ Department of Traumatology Semmelweis University Budapest Hungary; ^8^ FIFA Medical Centre of Excellence Budapest Hungary; ^9^ Department for Orthopaedics and Traumatology, Kepler University Hospital GmbH Johannes Kepler University Linz Linz Austria; ^10^ Department of Health and Rehabilitation, Institute of Neuroscience and Physiology, Sahlgrenska Academy University of Gothenburg Gothenburg Sweden; ^11^ Sportrehab Sports Medicine Clinic Gothenburg Sweden; ^12^ University Department of Orthopaedic Surgery and Traumatology, Head Knee Surgery and DKF Head of Research Kantonsspital Baselland Bruderholz Switzerland; ^13^ Department of Computer Science and Engineering Chalmers University of Technology Gothenburg Sweden; ^14^ Department of Orthopaedics Sahlgrenska University Hospital Mölndal Sweden

**Keywords:** artificial intelligence, causal inference, data analysis, machine learning, methods

## Abstract

While the magnitude and types of data available to orthopaedic researchers are steadily growing, standardized and efficient data management workflows for orthopaedic research using artificial intelligence (AI) are currently lacking. This work introduces essential principles and best practices for planning, collecting, storing, processing, labelling and governing data in AI‐based orthopaedic research. The various domains of available data quality guidelines for medical AI research are reviewed and discussed in terms of their adaptability to orthopaedic research datasets. In addition, future areas of improvement, such as registry development, the potential of synthetic data and gradual transition to continuous data streams for AI applications, are outlined.

**Level of Evidence:**

Level V.

**Abbreviations**
ACL: anterior cruciate ligament
AI: artificial intelligence
CT: computed tomography
DAG: direct acyclic graph
EDA: exploratory data analysis
EHR: electronic health record
FAIR: findable, accessible, interoperable and reusable
GDPR: General Data Protection Regulation
GRDI: Guidelines for Research Data Integrity
HIPAA: Health Insurance Portability and Accountability Act
LLM: large language model
ML: machine learning
MRI: magnetic resonance imaging
NLP: natural language processing
PRO: patient‐reported outcome
ROM: range of motion
RTS: return to sport
TRAIN: Trustworthy and Responsible AI Network

## INTRODUCTION

Proficiency in data management is a fundamental skill to acquire for all orthopaedic and medical researchers involved in the analysis of complex and heterogeneous datasets. Previous literature [28, 29, 32, 45, 47, 50] underscores the resource‐intensive nature of medical research implementing artificial intelligence (AI) and presents different challenges associated with the implementation of machine learning (ML), natural language processing (NLP) and generative AI for the analysis of medical data. The performance of AI systems depends not only on model architecture and implementation characteristics but also on data quality and structure. Due to variation in domain‐specific requirements for data collection across medical specialties, orthopaedic researchers should consider the context‐specific demands of data types, data volume and data quality for specific projects.

A structured, complete, relevant and high‐quality dataset enhances the practical utility and reliability of the AI system and is used for model training, optimization and validation. Conversely, incomplete, nongeneralizable or irrelevant data compromises AI system performance, which in turn wastes resources and produces output with limited scientific and clinical relevance. While data collection and management practices may vary depending on study design, institutional and regional practices and regulations, the presented work aims to highlight best practices (Textbox 1) and key principles for efficient and high‐quality data management pipelines in AI‐based orthopaedic research.

Textbox 1. **Best practices for data management in AI‐based orthopaedic research.**

- Use direct acyclic graphs (DAGs) early in the data management pipeline to visualize and assess the causal structure of the proposed dataset.
- Implement standardized data collection protocols, validated data entry forms and data dictionaries to reduce errors during data entry.
- Base data annotation consensus on domain expertise, with clearly defined taxonomies for labelling specific to the qualitative and quantitative aspects of orthopaedic data.
- Train researchers in efficient data transformation between wide‐format and long‐format data to optimize data for different analyses.
- Apply structured quality frameworks to evaluate the data quality of the collected information based on dimensions relevant to orthopaedic research.
- Conduct exploratory data analysis (EDA) to assess the completeness and balanced representation of patient subgroups within the dataset and thereby detect sources of bias and error early in the project.
- Ensure compliance with ethical and regulatory policies to protect sensitive patient information throughout the research pipeline.

## A GENERAL DATA MANAGEMENT WORKFLOW FOR AI‐BASED ORTHOPAEDIC RESEARCH

**Figure 1:**
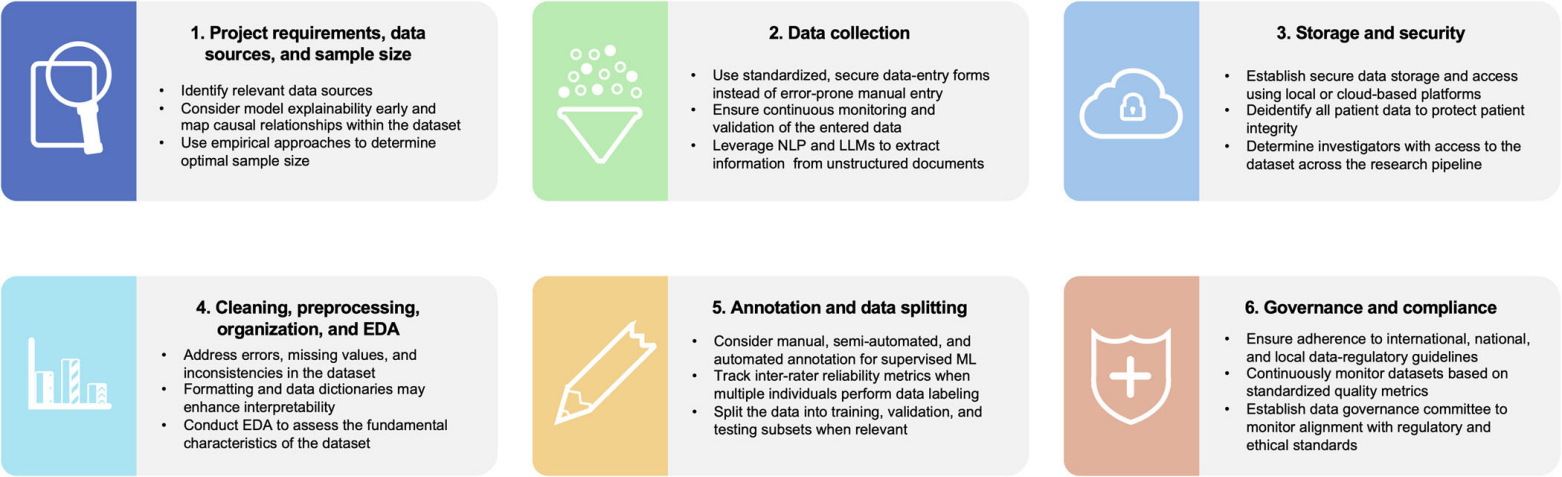
Schematic illustration of the general stages of data management for AI‐based orthopaedic research. EDA, exploratory data analysis; LLM, large language model; ML, machine learning; NLP, natural language processing.

Following standardized data‐management principles helps identify and minimize downstream errors throughout the AI research lifecycle. When implemented correctly, a data management plan considers factors with a potential impact on model performance prior to data collection and is monitored continuously throughout the entire project lifecycle. While data management workflows should always be designed and evaluated with the requirements and aims of the specific project in mind, the following steps serve as a general guide for data management in AI‐based orthopaedic research (Figure 1).

### Determining project requirements, data sources and sample size

The initial step in data management is to identify all potential variables and data sources required to develop a relevant and robust model to address the research question. Potential sources of data for orthopaedics include, but are not limited to, electronic health records (EHRs), subjective and objective clinical assessment data, medical imaging databases, orthopaedic registries, clinical trial data, wearable devices and unstructured clinical notes. Early consideration of data relationships and confounders is crucial. The resulting AI system must generate accurate, interpretable and clinically actionable predictions. Causality in medical research is the ability to reliably determine whether and why treatments cause positive or harmful effects. In orthopaedics, understanding the causal structure of data is particularly challenging because patient outcomes emerge from complex, time‐dependent interactions among patient‐related, anatomical, physiological and treatment‐specific factors [12]. Determining which of these relationships are truly causal and supported by sufficient evidence typically remains an area of ongoing methodological refinement and debate. As such, explicit discussions about the assumed causal structure are essential parts of study design and interdisciplinary collaboration and should influence data collection, data structure and subsequent analytical steps.

**Figure 2:**
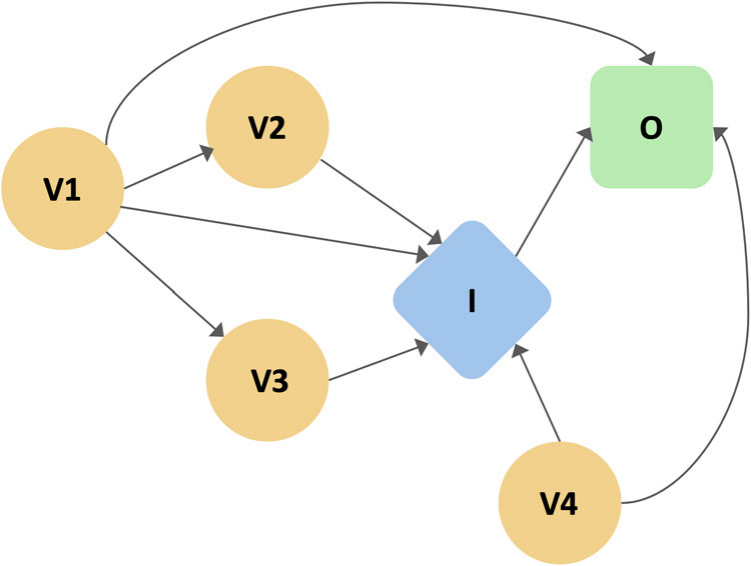
Schematic illustration of a directed acyclic graph (DAG) to visually represent assumed relationships between variables (yellow spheres V1‐4), interventions (blue diamond I) and outcomes (green rounded rectangle O) for causal modelling.

Direct acyclic graphs (DAGs) provide a visual approach to assess the causal structure of the proposed dataset (Figure 2) at an early stage in the data management pipeline and may clarify the need to adjust for confounders to assume unbiased interactions [33]. Additionally, DAGs may help pinpoint additional information to measure and collect for improved explainability of the final AI system. Consequently, a causal data management approach potentially enhances human interpretability while prioritizing inferential learning over spurious correlations within the queried data [12]. While the sample size of datasets to train and evaluate AI systems varies across use‐cases and datasets [5, 34], an empirical approach to sample size estimations is recommended for the development of orthopaedic AI systems, including domain‐specific generative models [13, 18, 24]. A priori sample size calculation may be advantageous in terms of increasing the explainability, robustness and overall utility of the final model, while averting wasteful efforts and resource allocation in the event of an insufficient magnitude of available data.

### Data collection

Once relevant data sources are identified, data must be collected using standardized protocols to ensure consistency and quality. The widespread use of spreadsheets for data management in medical research introduces several potential shortcomings and sources of error in data management for AI research in medicine [2, 7]. Data entry into spreadsheets is typically conducted manually, where formatting and human error may often be introduced. Without the validation of data at entry, introduced errors typically remain undetected until later stages of data analysis, jeopardizing the integrity of research findings. The use of systems with secure data entry forms, with labels and validated scales and ranges for the entered values, is therefore encouraged in every research scenario.

Generally, errors in data collection can be reduced by (1) determining specific variables in advance, (2) designing a suitable data‐entry framework, (3) pilot‐testing the data‐entry system before starting research, (4) prospective monitoring of the collected data for consistency and quality throughout the data collection process and (5) a final validation of the collected data based on the range of expected values [22, 36]. The increasing digitalization of medical reports and patient records that capture clinically relevant qualitative and quantitative information, enabling the centralization of such data in large‐scale clinical repositories and integration with existing radiological datasets and patient registries. NLP and large language models (LLMs) provide new semiautomated and automated methods for clinical information retrieval from unstructured medical reports and datasets [40, 41, 48, 49].

### Storage and security

Secure storage of the collected research data involves choosing appropriate data storage solutions (e.g., cloud‐based platforms, centralized local databases) and implementing access controls to protect sensitive patient information. The removal of identifiable and sensitive patient information (ensuring that patients cannot be reidentified) from such datasets is an important step for the collection and subsequent sharing of data between interdisciplinary stakeholders involved in AI‐driven medical research. Pseudonymization, anonymization and deidentification of medical datasets are resource‐intensive, but essential technical measures to ensure data security. A recent study highlights the use of an LLM‐based clinical data deidentification pipeline using zero‐shot inference, with a 99.24% success rate for the removal of sensitive medical information from unstructured clinical text data [43], which suggests that automated data anonymization pipelines may facilitate safe and efficient data deidentification for medical research in the near future.

In the context of medical data, privacy refers to the methods that ensure the recording and storage of data in a manner that protects personal integrity, while security refers to the measures used to prevent unauthorized access and modification to existing datasets. Healthcare data comprise a special category of sensitive personal information stored in centralized data repositories and are subject to cybersecurity and privacy‐related breaches [1, 9]. Consequently, it is essential that data collection and processing pipelines for AI systems are compliant with national and international regulatory guidelines to safeguard patient integrity and prevent potential legal consequences for healthcare and research organizations. At the most fundamental level, data management for AI‐based orthopaedic research requires compliance with the findable, accessible, interoperable and reusable (FAIR) principles [44], which ensure that the dataset used for the implementation of the project is FAIR. The specific regional legal requirements of AI‐based medical research are subject to ongoing development and exceed the scope of this text. However, the general characteristics of datasets compliant with data protection policies include transparency (informed consent, ethical approval, publicly available communications), anonymization and the ability of subjects to opt out of inclusion in the training dataset.

### Cleaning, preprocessing, organization and exploratory analysis

Raw data often contains errors, missing values and inconsistencies that can negatively impact AI model performance. This step involves cleaning the data by identifying and handling entry errors, handling missing data (e.g., imputation or removal) and transforming the data into a suitable format for machine readability. Preprocessing may include tasks like normalization, feature scaling and handling outliers.

**Figure 3:**
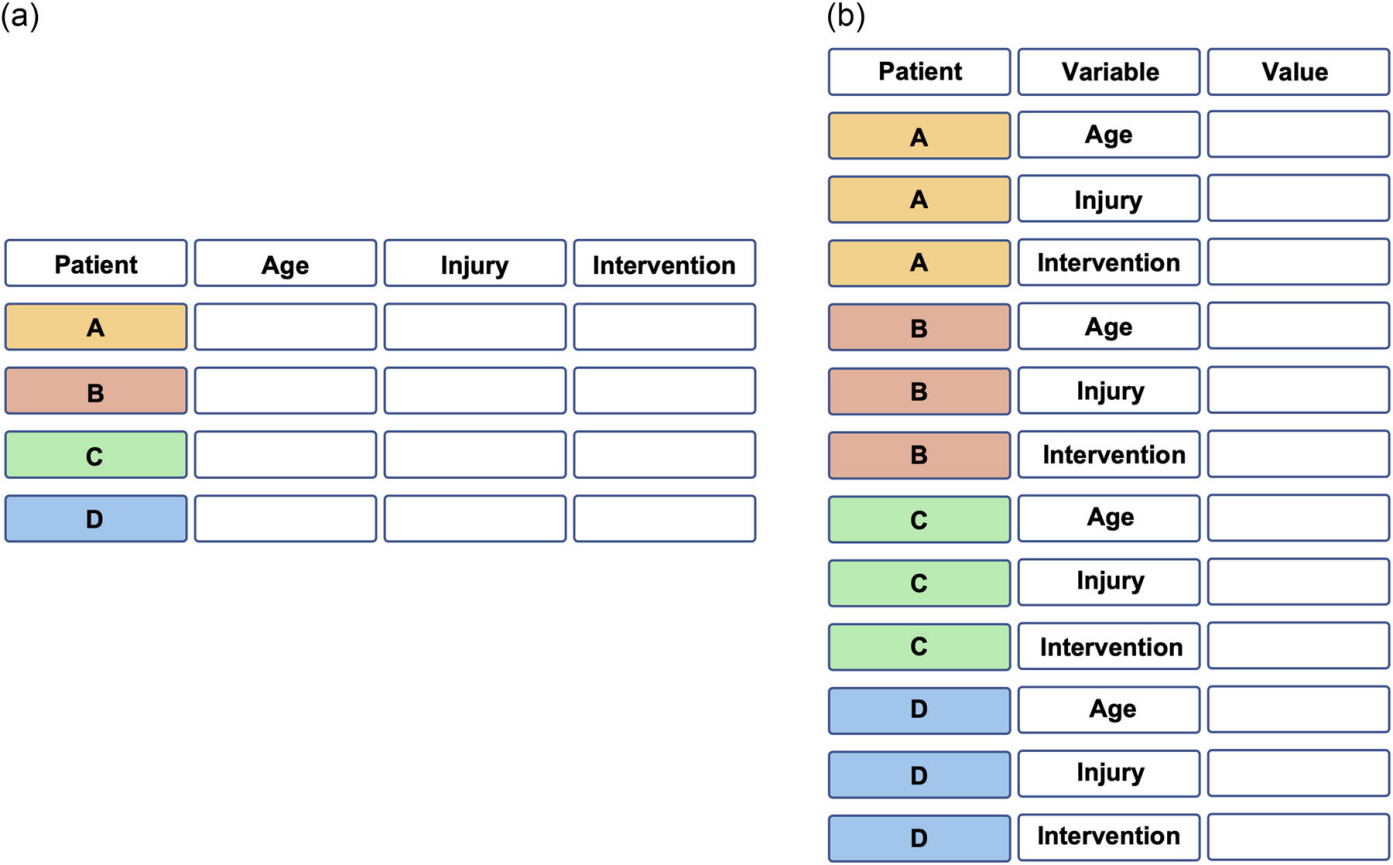
Schematic illustration of (a) wide‐ and (b) long‐format data tables with respect to the arrangement of data in a hypothetical dataset that contains values patient identifier, age, injury and intervention.

Further consideration of data organization is essential for implementing efficient data management and analysis pipelines. The research project may require different formatting of the collected data with regard to human‐ versus machine‐readability. While colour‐coding variables and values may be attempted with the aim of enhanced human‐readability, this practice is not beneficial from the perspective of machine readability, which requires other practices to highlight the desired variables or groupings within the raw dataset. Instead, the creation of data dictionaries [23] for variable names and attributes is recommended to enhance the human interpretability of variables captured in large datasets. Most data management platforms enable the collection of data in two‐dimensional data tables, which typically use a wide or a long format, each with several advantages and disadvantages based on the intended purpose. Wide data tables assign one row to one subject, where each column corresponds to a separate variable. A wide data format is favourable for human‐readability, enhancing visual interpretability, as well as low‐level computational tasks like summarization and statistical comparison between two different variables. In contrast, wide data tables are disadvantageous from the aspect of machine‐readability, as they scale poorly to the addition of new variables (potentially compromising existing calculation pipelines), store variable names as data and are suboptimal for large‐scale datasets used for ML applications. Long data tables assign one row to each new observation/variable for a specific subject, with each column corresponding to a specific property of a variable (meta‐data). A long data format is advantageous with regard to machine‐readability, facilitating tasks such as statistical modelling, complex data visualization, time‐series analysis of repeated measures and ML tasks in a scalable manner. However, the fragmented structure of long tables hampers human interpretability at a glance (by appearing as repetitive data entries for a single subject). Rather than using a single, static format, researchers should learn to convert rapidly between formats within their preferred pipeline to optimize data for specific tasks (Figure 3).

Exploratory data analysis (EDA) conducted at this stage of the data management workflow may be beneficial to assess the completeness and distribution of the collected data, to verify that patient subgroups are represented equally and to identify remaining sources of error in the dataset prior to analysis [31].

### Annotation and data splitting

Annotation refers to the process of marking or describing relevant entities or features in raw data (e.g., outlining anatomical structures in an image or highlighting text spans in a clinical note). Labelling, in contrast, assigns categorical or numerical values to those annotated entities (e.g., ‘fractured’ vs. ‘non‐fractured’, or grading the severity of cartilage damage). Depending on the experimental design, it may be essential to clearly define a detailed taxonomy of labels for annotating the collected dataset. Comprehensive and standardized labelling of unstructured data, such as radiology reports, medical images and physical therapy progress reports, enhances the granularity of the overall dataset and grants a deeper understanding of the variables associated with the studied pathology or injury. Consequently, thoroughly annotated datasets provide access to specific predictive diagnostic entities and outcomes, which are likely to improve model performance, clinical utility and interpretability in the right context. For supervised ML tasks, data annotation and labelling are essential. This involves assigning meaningful labels to the data, such as classifying images as ‘fractured’ or ‘non‐fractured’, or labelling patient outcomes as ‘successful’ or ‘unsuccessful’. The contribution of domain expertise in orthopaedics is warranted for accurate and consistent annotation standards to avoid variability across studies, especially when it comes to labelling medical imaging parameters and qualitative clinical data.

Before training an AI model, the dataset is typically split into three subsets: training, validation and testing sets. The training set is used to fit the model parameters, while the validation set is employed during model development to fine‐tune hyperparameters, monitor learning progress and prevent overfitting or selection bias [14]. Finally, the testing set is reserved for the final evaluation of model performance and generalization on previously unseen data. There is no clear consensus regarding the most optimal train‐test data split ratio. Typical ratios ranging between 50:50 and 90:10, ratio selection has a direct impact on model performance and should be considered for each research project to improve predictive accuracy [30].

### Governance and compliance

Continuous data governance and regulatory compliance throughout both the research stage and subsequent clinical development and deployment lifecycle are essential to monitor the quality, trustworthiness and safety of AI systems [47]. This involves establishing and enforcing policies and procedures for data handling, security, privacy and ethical considerations. Regular audits and adherence to regulations like Health Insurance Portability and Accountability Act (HIPAA) and General Data Protection Regulation (GDPR) are essential to maintain patient trust and research integrity. In practice, data governance requires the surveillance of key metrics such as data drift (changes in patient populations or surgical techniques over time), prediction drift (shift in model output), bias rate, performance decay (in terms of accuracy, sensitivity, specificity) and regular quality audits for data entry and labelling (e.g., for evolving classification systems) [39]. Orthopaedic registries should implement governance frameworks for multi‐institutional data linkage and consistency, temporal validation and scenarios where outcome definition and standards of care change over time. For example, an AI model predicting prosthetic joint infection risk in a national registry may require recalibration when new national antibiotic protocols (data drift) are introduced, retraining to address underperformance in geriatric patients (bias detection) and a review of predictive accuracy after the adoption of robotic‐assisted surgical techniques (performance decay). Practical collaborative consortiums such as the Trustworthy and Responsible AI Network (TRAIN) have recently been established to facilitate the responsible adoption of AI across different organizations [8]. Key data management steps from an AI governance perspective include the continuous monitoring of real‐world datasets for quality, safety, transparency, sources of bias, scalability and performance shift over time [42].

## DATA QUALITY REQUIREMENTS

It is well established that the quality of ML models hinges on the quality of data used to train and test ML models for specific tasks. Consequently, it is important to clarify the aspects of data that may ultimately influence model quality, to help researchers consistently fulfil well‐defined requirements for AI applications in an orthopaedic research context.

Currently, there is a shortage of comprehensive frameworks to help improve the integrity and quality of data for orthopaedic research. Adherence to the Guidelines for Research Data Integrity (GRDI) framework [27] may serve as a general reference for orthopaedic researchers to standardize data quality across research pipelines. However, AI‐based projects require further awareness of the ongoing development of guidelines to improve reporting standards and quality specific to preclinical, translational and clinical AI research [3, 19, 22, 36]. From the perspective of data management, the FUTURE‐AI guidelines emphasize clearly defining sources of data variation, data representativeness and data‐related risk management in the context of the research project [22]. Additionally, the recently published METRIC framework [36] proposes five key data quality dimensions to improve the robustness, interpretability and trustworthiness of AI models developed with data trained on a specific dataset. The METRIC framework defines the (1) measurement process, (2) timeliness, (3) representativeness, (4) informativeness and (5) consistency of datasets as key characteristics from a quality assessment perspective. While the presented dimensions may not present an exhaustive set of quality domains for every orthopaedic research scenario, awareness of essential data quality domains may help improve the quality of training and testing data, as well as the subsequent performance of AI models (Table 1). Together, these frameworks form a hierarchical continuum: GRDI defines overarching integrity principles, FUTURE‐AI adapts them to the healthcare AI context, and METRIC provides practical operational criteria for assessing dataset quality in medical AI research.

**Table 1:** Data quality properties for AI‐based orthopaedic research adapted from clusters defined by the METRIC framework [36].

Quality dimension	Definition	Examples
Measurement process		
Device errors	Technical inaccuracies or imprecision in measurement tools	Calibration errors in radiographic joint angle measurements Inconsistent CT and MRI image quality Force plate inaccuracies in gait analysis Variability in objective knee laxity measurements for ACL injury
Human‐induced errors	Errors introduced through human data collection or interpretation	Ambiguity in fracture classification Variation in the interpretation of clinical exam findings and surgical outcomes Inter‐observer variability in the correct localization of surgical landmarks Data labelling errors in medical records and unstructured sources
Completeness	Extent of missing values and representation of relevant variables within the dataset	Incomplete PRO scores due to attrition or loss‐to‐follow‐up Inclusion of all relevant demographic, injury‐related and surgical variables Inconsistent reporting of comorbidities and confounders Discrepancies in rehabilitation protocol description and standardization
Source credibility	Reliability of data sources	Standardized data collection protocols across single‐centre versus multi‐centre research Alignment in the reliability of data sourced from academic versus community versus private hospitals Reliability and validity of patient and clinician‐reported outcomes Clinical relevance of registry data versus randomized controlled trial data
Timeliness		
Age	The relation between the creation date and the usage date	Obsolete data from outdated procedures no longer represent the standard of care Distributional shifts in patient demographics over time Changes in device/implant technology and design over time
Currency	How up‐to‐date the data is	Use of up‐to‐date injury classification systems Rehabilitation protocols that conform to the most recently accepted guidelines Updated complication reporting standards
Representativeness		
Variety	Breadth of demographics and data sources	Patients included across paediatric, adult and geriatric populations Patient‐sex representation in joint preservation/replacement outcomes Ethnic diversity in anatomical, physiological and genetic variables Representation of rare versus common injuries/phenotypes
Depth of data	Sufficient data volume overall and within subpopulations	Adequate overall sample size for reliable assumptions Sufficient granularity of patient, injury, surgical and rehabilitation variables (e.g., meniscus tear location, chondral injury grade, objective knee laxity magnitude, osteoarthritis stages) Longitudinal data spanning short‐ and long‐term follow‐up intervals
Target class balance	Appropriate representation of outcome classes	Balanced representation of treatment success and failure Proportional inclusion of simple and complex disease/injury phenotypes
Informativeness		
Understandability	Clarity and unambiguity of data	Standardized terminology for surgical techniques and variables Consistent use of injury/disease classification and coding systems Clarification of primary and revision surgery, and surgical timing in relation to injury incidence Clear and standardized definitions of treatment success and failure
Redundancy	Duplication of information	Overlapping subjective outcome scores that measure the same phenomenon Duplicate patient entries
Informative missingness	Whether missing values carry meaningful information	Patients lost to follow‐up due to suboptimal outcomes or attrition Lack of advanced imaging to assess relevant anatomic variables prognostic of disease/injury outcome Incomplete rehabilitation data for non‐compliant patients
Feature importance	Value added by specific data elements	Relevance of anthropometric data and functional phenotypes for joint replacement outcomes Importance of bone quality measures for fracture fixation Assessment of modifiable anatomical variables that may impact treatment outcomes
Consistency		
Rule‐based consistency	Adherence to format and structure rules	Standardized reporting of joint range of motion and laxity Consistent radiographic measurement techniques Consistent application of fracture classification systems Standardized complication reporting Consistent application of RTS criteria
Logical consistency	Logical soundness without contradictions	Alignment and content validity of subjective and objective outcome measures Consistency between clinical assessment and imaging findings Agreement between functional outcome measures and decision to RTS
Distribution consistency	Similar statistical properties across subpopulations	Comparable variance in variables measured across different institutions Similar distributions of complications across surgeon experience levels Consistent dataset completeness patterns and loss to follow‐up rate across demographic groups and institutions

Abbreviations: ACL, anterior cruciate ligament; AI, artificial intelligence; CT, computed tomography; MRI, magnetic resonance imaging; PRO, patient‐reported outcome; RTS, return to sport.

## FUTURE DIRECTIONS

### Improving the quality of orthopaedic registry data

While registries are frequently queried sources of representative and high‐quality injury and disease‐specific data in orthopaedic research, several inherent limitations hamper the clinical relevance and interpretability of registry data for AI‐based research [21]. In particular, inconsistencies in the coding of diagnoses, lack of granularity with respect to variables associated with the injury or pathology of interest, information about physical therapy protocols, and incomplete PROs over time due to patient attrition render the collected data of suboptimal quality for generalizable and clinically relevant AI system development [21]. The utility of next‐generation orthopaedic registries for AI applications may therefore require a more comprehensive and broad inclusion of data regarding patient demographics, injury‐related factors, surgical variables and information pertinent to physical therapy and rehabilitation. Furthermore, an improved understanding of the objective role of anatomical factors on patient outcomes requires the adoption of standardized assessments on radiologic imaging modalities [52]. An AI‐assisted approach for the assessment of quantitative and qualitative structural imaging biomarkers [15, 25, 37] may reduce human bias and systematic error, as well as the resource‐intensive nature of imaging data collection, facilitating their future inclusion in patient registries.

While PROs are frequently collected across registries and serve as quantitative measures of subjective functional outcomes in orthopaedic research, several limitations associated with their use may render them suboptimal for inclusion in AI systems. In their current state, the inclusion of PROs model input and predicted output may be uninformative due to fragmented collection and missing data in the training set, patient attrition due to inadequate infrastructure for PRO collection, inappropriate PRO selection and lack of validation for the target population [4, 20, 51]. Future incorporation of data collected from digital health technologies in patient registries presents a potential opportunity for improvement to address shortcomings in the measurement of patient‐centred clinical outcomes [26]. Sensor‐based technologies and electronic questionnaires augmented with LLM‐based conversational agents may improve dataset quality by helping clinicians and orthopaedic researchers determine which subjective and objective health metrics are associated with clinically meaningful patient‐centric endpoints, in a pragmatic and data‐driven manner [4, 26, 29].

### Synthetic data

Synthetic data is defined as algorithmically generated information that preserves the inherent statistical characteristics, relationships and distributions of real‐world data, without additional collection of real‐world information. Synthetic data can be generated using a variety of methods, ranging from more traditional statistical modelling‐based methods to generative adversarial networks, variational autoencoders and diffusion models [35, 50]. Potential benefits for use in orthopaedic research include reduced security risks and ethical compliance requirements, and therefore facilitated data‐sharing across multiple institutions and stakeholders. Furthermore, synthetic data can be used to address imbalances in datasets between subgroups, and may yield additional data in the setting of small patient populations, with an otherwise insufficient sample size to permit adequate testing and validation splits [10, 16, 17].

In a more pessimistic scenario, widespread generative AI use may contaminate existing datasets with synthetic information. This could compromise the integrity and reliability of real data. The downstream effect of the phenomenon where ML models ingest the synthetic output generated by other (or their own) ML models has been termed AI autophagy [46] and may inadvertently deter sustainable and ethical medical AI development [11].

### Static versus continuous data streams

It is likely that agentic AI workflows will catalyze a transition from static to continuous data streams, which will enable AI systems to gain experience through a dynamic and continuous process without the need for human intervention to update training and validation data [38]. While the current standard is to train medical AI systems on cross‐sectional, historical patient data based mainly on human observations that are subject to variability and bias, the aim of a continuous data approach is to gain further insight regarding orthopaedic conditions and intervention effects based on the continuous monitoring of complex patterns, granular domain‐specific data and increased patient engagement [6, 38].

## CONCLUSION

This guide aims to empower orthopaedic researchers to implement robust, transparent and reproducible AI pipelines. As the landscapes of medical AI research, data governance and legal requirements continue to evolve, orthopaedic researchers should familiarize themselves with fundamental data management skills, including causal learning, annotation, data processing, quality assessment, EDA and data governance. Taken together, advancements in registry data quality, the responsible use of synthetic data, and the transition toward continuous data streams represent complementary pillars of a next‐generation orthopaedic AI ecosystem. Integrating these developments will enable more comprehensive, representative and dynamically updated datasets—laying the groundwork for AI systems that continuously learn from real‐world evidence while maintaining clinical relevance, transparency and trustworthiness. Our goal is to empower orthopaedic researchers to implement robust, transparent and reproducible AI pipelines.

## AUTHOR CONTRIBUTIONS

All listed authors have contributed substantially to this work. Statistical analysis, review of the literature and primary manuscript preparation were performed by Bálint Zsidai, Felix Oettl, James A. Pruneski and Yinan Yu. Editing and final manuscript preparation were performed by Bálint Zsidai, Felix Oettl, James A. Pruneski, Gergely Pánics, Philipp W. Winkler, Eric Hamrin Senorski, Michael T. Hirschmann, Yinan Yu, Robert Feldt and Kristian Samuelsson. All authors have read the final manuscript and given final approval for the manuscript to be published. Each author consented to be accountable for all aspects of the research in ensuring that questions related to the accuracy or integrity of any part of the work are appropriately investigated and resolved.

## CONFLICT OF INTEREST STATEMENT

Michael T. Hirschmann is a consultant for Medacta, Symbios and Depuy Synthes and is Editor‐in‐Chief for Knee Surgery Sports Traumatology Arthroscopy (KSSTA). Kristian Samuelsson is a member of the board of directors for Getinge AB and is a medical technology advisor for Carl Bennet AB. Robert Feldt is Chief Technology Officer and founder of Accelerandium AB, a software consultancy company. Philipp W. Winkler is employed as a web editor for KSSTA.

## ETHICS STATEMENT

The authors have nothing to report.

## Data Availability

Data sharing is not applicable to this article, as no datasets were generated or analysed during the current study.
